# Anthropometric Measurements and Laboratory Methods for Pregnancy: An Update Review to Evaluation of Body Composition

**DOI:** 10.1007/s13668-024-00597-x

**Published:** 2025-01-08

**Authors:** Yasemin Açar, Eda Köksal

**Affiliations:** 1 Department of Nutrition and Dietetics, Ondokuz Mayıs University, Samsun, Türkiye; 2 Department of Nutrition and Dietetics, Gazi University, Ankara, Türkiye

**Keywords:** Anthropometric measurements, Body composition, Gestation, Laboratory methods, Pregnancy

## Abstract

**Purpose of Review:**

The aim of this review is to summarize and put into context the current evidence on anthropometric measurements and laboratory methods used in the evaluation of body composition in pregnancy, in the light of current studies.

**Recent Findings:**

Protecting women's health during pregnancy, childbirth and the postnatal period is important for maternal health. Pregnancy is a critical period during which the female body undergoes significant changes to support fetal growth and development. Maternal changes in body composition are associated with fatal development and maternal health during pregnancy. Anthropometry is a simple, reliable, and low-cost method that can be applied globally in primary care for evaluating maternal nutritional status. Maternal anthropometry is important in predicting various complications that may occur during pregnancy, such as intrauterine growth retardation and the risk of low birth weight. In this context, anthropometric measurements such as body weight, skinfold thickness, and middle upper arm circumference, and laboratory methods such as body water, body density, bioelectrical impedance analysis, ultrasound, dual-energy X-ray absorptiometry, and magnetic resonance imaging are frequently used in estimating the body composition of pregnant women. In addition to body weight gain monitoring, it is possible to determine the body composition of a pregnant woman by using different anthropometric measurements and the proposed equations.

**Summary:**

Accurate evaluation of anthropometric measurements and body composition in pregnant women is important in protecting the health of the mother and baby and in the early diagnosis of complications that may occur during pregnancy.

## Introduction

Changes in maternal body composition during pregnancy are closely related to fetal and maternal health [1]. For this reason, it is important to evaluate and monitor the body composition of the pregnant woman. Methods used in the evaluation of body composition; divide the body into compartments derived from the theoretical estimate of adipose tissue. The two-compartment model divides the body into adipose tissue and lean tissue; the three-compartment model separates the lean tissue compartment into body water and a mineral-protein combination [2]. By these models, various anthropometric measurements and laboratory methods, especially gestational weight gain, may be used for assessing body composition. Additionally, it is possible to evaluate the body composition of the pregnant woman through some specific equations with different anthropometric measurements [3].

### Anthropometric Measurements

Anthropometric measurements, particularly body weight, skinfold thickness, and middle upper arm circumference (MUAC), have been widely used to predict changes in body composition during pregnancy.

### Evaluation of Body Weight during Pregnancy

Phenotypic and physiological changes during pregnancy include enlargement of the placenta along with increases in maternal and fetal weights and gestational weight gain, as well as changes in amniotic fluid [4]. Approximately 2 L of blood and 2 L of extracellular fluid accumulate in the maternal tissues, with an increase of approximately 2 kg in the uterus and breast tissue [5]. When fetal tissues consisting of the placenta, amniotic fluid, and fetus are evaluated; there is a linear increase in the placenta until the 24th week (~ 150 g), and in the third trimester, the placental weight reaches 600–700 g with a more linear increase. The weight of the fetus reaches 1 kg by the 28th week of pregnancy and then, the fetus gains approximately 2.5 kg in the remaining 12 weeks of pregnancy [1, 6, 7].

The fetus, the placenta, amniotic fluid, uterus and breasts are among the components that cause weight gain during pregnancy. Body weight components and changes during pregnancy are shown in Table 1 [8].

**Table 1:** Body weight components and changes during singleton pregnancy

Tissues and Fluids	Total Body Weight Gain (g)			
	10th week	20th week	30th week	40th week
Fetus	5	300	1500	3400
Placenta	20	170	430	650
Amniotic fluid	30	350	750	800
Uterus	140	320	600	970
Breasts	45	180	360	405
Blood	100	600	1300	1450
Extravascular fluid	0	30	80	1480
Maternal stores (fat)	310	2050	3480	3345
**Total**	**650**	**4000**	**8500**	**12 500**

The International Institute of Medicine (IOM) guidelines aims to optimize maternal, fetal, and infant health outcomes and recommends that the mother achieves a healthy pre-pregnancy body weight. IOM gestational weight gain recommendations for singleton and twin pregnancies are presented in Table 2 [9].

**Table 2:** Gestational weight gains according to IOM during singleton and twin pregnancies

Pre-Pregnancy BMI Category	Pre-Pregnancy BMI (kg/m^2^)	Total Weight Gain Range (kg)	
		Singleton pregnancy	Twin pregnancies
Underweight	< 18.5	12.5–18	-
Normal	18.5–24.9	11.5–16	17–25
Overweight (Pre-obesity)	25.0–29.9	7–11.5	14–23
Obese*	≥ 30.0	5–9	11–19

^*^Includes all classes of obesity

For term deliveries between 37 and 42 weeks gestation, IOM recommend a total weight gain of 12.5–18 kg for underweight women (pre-pregnancy BMI: < 18.5 kg/m^2^), 11.5–16 kg for women with normal weight (pre-pregnancy BMI: 18.5–24.9 kg/m^2^), 7–11.5 kg for overweight women (pre-pregnancy BMI: 25–29.9 kg/m^2^), and 5–9 kg for women with obesity (pre-pregnancy BMI > 30 kg/m^2^) during singleton pregnancy. For twin pregnancies total pregnancy weight gain is 17–25 kg for women with normal weight, 14–23 kg for overweight women, and 11–19 kg for women with obesity [9]. Excessive gestational weight gain increases the risk of cesarean delivery and may be associated with an increased risk of abnormal glucose metabolism and hypertension. Additionally, infants are at risk of increased birth weight, macrosomia, impaired fetal growth, and preterm delivery [10–12]. After birth, mothers with excessive gestational weight gain; and obesity are at risk of obesity-related health outcomes, including type 2 diabetes, and cardiovascular disease [13].

In a study by Zhao et al., the effects of pre-pregnancy body mass index (BMI) and pregnancy weight gain on birth weight and whether weight gain meets IOM recommendations were examined. It has been observed that the probability of low birth weight and small for gestational age is higher in those with weight gain below the IOM recommendations, and a higher probability of macrosomic and large for gestational age births in those with weight gain above the IOM recommendations [14].

In 2016, INTERGROWTH-21, a multi-country project to extend the World Health Organization (WHO) Child Growth Standards to the fetal and neonatal period, was initiated. In addition to fetal and neonatal growth standards, gestational weight gain standards have been developed with INTERGROWTH-21 for women with normal pre-pregnancy BMI. With these standards, weight gain can be followed according to the week of gestation during pregnancy, and complications that may occur can be predicted in advance. Studies are still ongoing on standards for overweight women [15, 16].

### Skinfold Thickness

Body composition evaluation by measuring skinfold thickness is non-invasive and suited for field research because no specific equipment is required. Skinfold thickness protocols usually comprise measurements at 4–7 sites (e.g., triceps, biceps, subscapular, suprailiac, thigh, and calf) [1]. The sum of skinfold thickness measurements from specific areas of the body is used to estimate total body subcutaneous fat. Skinfolds, along with maternal weight and circumferences (e.g., wrist circumference, MUAC) have been used generate prediction equations for calculating % body fat in pregnant women [17–22]. These skinfold prediction equations are based on maternal age and race. Most equations are old and their validation studies do not include many women with obesity (excluding a study conduct by Kannieppan et al. [21]); therefore, the relevance of skinfold equations for % fat in women with obesity is unknown [1].

There is evidence that skinfold thickness starts to increase in the first trimester and continues in the second and third trimesters. Skinfold thickness changes due to increased hydration of the extracellular components of connective tissues due to hormonal changes. There are some limitations to estimating the adipose tissue and body fat percent from skinfold thickness measurement. During pregnancy, it is difficult to obtain the correct skinfold thickness from the same area due to the expansion and stretching of the skin [3, 23]. Skinfold thickness measurement is subjective and therefore, requires extensive training to ensure reliability. The increase in skinfold thickness may differ according to the area measured during gestation [1].

Paxton et al. developed and validated equations to predict the adipose tissue change from week 14 to week 37 [17]. Similarly, Huston Presley et al. developed and validated an equation for calculating adipose tissue mass from skinfold thickness measurements and body weight at 30 weeks of gestation [18]. However, these equations are for certain gestational periods and are not suitable for use at other times of pregnancy. Therefore, it is often preferred to use skinfold thickness measurements instead of adipose tissue and lean tissue estimations. These equations that using skinfold thickness measurements in pregnancy are summarized in Table 3.

**Table 3:** Body composition equations using skinfold thickness measurements in singleton pregnancy

References	Body composition	Anthropometric Equations
Paxton [17]	Fat tissue change between 14–37 weeks	Fat tissue change (kg) = 0.77 (weight change, kg) + 0.07 (change in thigh skinfold thickness, mm)—6.13 Fat tissue change (kg) = 0.84 (weight change, kg) – 6.49
Paxton [17]	Body fat mass at term (37 weeks)	Fat mass (kg) = 0.40 (weight at 37 weeks, kg) + 0.16 (biceps skinfold thickness at 37 weeks, mm)—0.15 (thigh skinfold thickness at 37 weeks, mm)—0.09 (wrist circumference at 37 weeks, mm) + 0.10 (pre-pregnancy weight, kg)—6.56
Huston Presley [18]	Body fat mass at 30 weeks	Fat mass, kg = 0.33529 (weight, kg) + 0.65664 (triceps skinfold thickness, mm) ‒ 0.4373 (subscapular skinfold thickness, mm) + 0.43461 (suprailiac skinfold thickness, mm)—13.0538
Kannieappan [21]	Percent of body fat between week 10–20 (BF% in women with overweight or obesity)	BF% = 12.7 + (0.457 × triceps SFT) + (0.352 × subscapular SFT) + (0.103 × biceps SFT) – (0.057 × height) + (0.265 × arm circumference)
Durnin [20]	Body density	DB = 1.1581 – 0.0720 (log of the sum of TSF + BSF + SSSF + SISF)
Siri [22]	Percent of body fat	Fat % = [(4.95 / Db)—4.5] × 100

*Kg* kilogram, *mm* milimeter, *BF* body fat, *SFT* skinfold thickness*, DB* body density, *TSF* triceps skinfold, *BSF* biceps skinfold, SSSF subscapular skinfold, *SISF* suprailiac skinfold, *Db* body density

In a study conducted by Kretzer et al., it was aimed to determine the relationship between the amount of maternal adipose tissue and various anthropometric measurements (body weight, height, MUAC, calf and neck circumference, triceps and subscapular skinfold thickness), and ultrasound measurements (visceral, subcutaneous and total adipose tissue)). It has been stated that the best-correlated measurements in determining maternal adipose tissue are MUAC and subscapular skinfold thickness, and these measurements are low-cost and applicable measurements to provide effective and reliable estimation of visceral and subcutaneous adipose tissue, especially in low- and middle-income countries [24].

### Middle Upper Arm Circumference

Middle upper arm circumference is often used to measure lean mass. It is a measurement of the upper-arm circumference at the midpoint between the olecranon and acromion processes. Because the arm contains both subcutaneous adipose tissue and muscle tissue, changes in the MUAC may reflect a change in muscle mass, subcutaneous adipose tissue, or both. It is estimated that changes in the MUAC of individuals with low subcutaneous fat tissue reflect the change in muscle mass [25]. Various cut-off points are used by different institutions for pregnant women. According to international organizations, MUAC < 23 cm carries a risk of growth retardation in the fetus, while MUAC < 21 cm is frequently used in emergency interventions [26].

In a systematic review examining the relationship between low MUAC and health outcomes in pregnancy within the scope of the Food and Nutrition Technical Assistance III Project (FANTA) funded by the US Agency for International Development (USAID), it was reported that low-maternal MUAC was associated with an increased risk of preterm birth, intrauterine growth retardation and small for gestational age [27]. It has been stated that since individuals in countries with low socioeconomic levels tend to have less amount of subcutaneous fat tissue, changes in MUAC may more easily reflect changes in muscle mass during pregnancy [28].

## Laboratory Methods

### Total Body Water and Hydrometry

During pregnancy, total body water (TBW) changes are highly variable. The principle of hydrometry is based on the use of stable isotopes (e.g., deuterium, and tritium) to estimate body water and lean tissue. Individuals must consume some amount of deuterium oxide (D_2_O), which is the isotope of water, and the body is expected to reach equilibrium in the TBW pool in (approximately 2–5 h). A urine (or saliva) sample is collected to calculate the amount of isotope present in the TBW pool. The advantages of using a deuterium isotope that is not harmful to pregnant women or the fetus include the use of newer, portable devices to measure isotope richness and the use of deuterium dilution in studies. New portable equipment for measuring isotope dilution (e.g., Fourier Transform Infrared Spectrometry), deuterium dilution is possible in field research. The disadvantages include that the measurement takes approximately 4–5 h, pregnant women may not want to consume the stable isotope and the hydration values obtained are specific to the populations [29].

The size of the body water pool is determined by the concentration of the isotope and is described as the following: [29].

- TBW (kg) = Dose of D_2_O (mg) / Amount of isotope determined in urine or saliva (mg/kg)

### Body Density

Body density can be estimated using hydrodensitometry (HD), known as underwater measurement, or air-displacement plethysmography (ADP). These methods cannot assess the body density of pregnant women independently of the fetus and pregnancy-related tissues. Estimation of body components by these methods is affected by changes in lean tissue density and composition throughout pregnancy. Although the initially increase in lean tissue is predominantly maternal tissue, as pregnancy progresses, the lean tissue changes are predominantly more fetal tissue [30].

Hydrodensitometry is the gold standard for measuring body density. Measuring body weight underwater is a non-invasive technique using Archimedes' principle. The weight of a submerged object is equal to the weight of the displaced water, and the following calculation method is used to calculate the weight [31].

- Body volume (L) = Body weight in air (kg)—Body weight underwater (kg) / Density of water (kg/L)

For the measurement of body volume using underwater weighing, individuals are immersed in water and asked to hold their breath after exhaling for 10 s [32]. As the underwater measurement is a non-invasive and sensitive method, it does not pose any risk to pregnant women. However, the disadvantages of this method include the difficulty experienced in the correct execution of the respiratory function because the procedure takes 2–3 min and it may be difficult for pregnant women to submerge in water, especially at the end of pregnancy [3].

### Air Displacement Plethysmography

ADP technology is a method for estimating body volume by measuring or estimating the thoracic gas volume, based on Boyle's law. With its increasing use in pregnancy, it is becoming a widely adopted body composition assessment method and it has potential for studies during pregnancy. BOD POD is an air displacement plethysmograph device that uses whole-body densitometry to determine body composition (body fat and lean mass) in adults and children [33].

ADP has advantages such as high level of safety and participant tolerance, allowing for repeated assessments during pregnancy. The methodology is simple to follow and allows for a quick measurement of body volume in under 5 min [1], but has limitation, BOD POD is expensive, requires equipment and is not portable for field researches. Obtaining true measurement of thoracic gas volume is difficult for pregnant women because during pregnancy, thoracic gas volume decreases by approximately 100 mL every trimester [34, 35]. If thoracic gas volume ignored, fat mass (FM) would be overestimated, especially in late pregnancy [1, 36].

### Bioelectrical Impedance Analysis

Bioelectrical impedance analysis (BIA) is an inexpensive and practical body composition measurement method for measuring body water and body composition. BIA is a non-invasive method based on assumptions in the electrical conductivity of biological tissues. The measurement is performed by sending a current to the body with the help of electrodes placed on the wrists and ankles [37, 38].

The electrical conductivity of the current varies according to the amount of water present in different biological tissues. Tissues with high water content, such as skeletal muscle, are more conductive than adipose tissue or bone tissues with lower water content. In pregnant women, in the early stages of pregnancy at (14 weeks); BIA was found to provide valid estimates of body water compared with the deuterium dilution method. However, it has been reported that BIA underestimates body water later in pregnancy at (32 weeks) [37].

BIA measurements, may be affected by fluid changes during the day. For this reason, it is important to standardize the time of the day in the BIA measurements [38]. The disadvantage of using BIA is that body water is affected by the ratio of intracellular fluid (ICW) to extracellular fluid (ECW) that changes throughout pregnancy. BIA devices estimate this ratio with regression equations developed by the manufacturers, but comparisons of these estimates with reference methods have not been reported [39, 40].

In a study conduct by Trindade et al., it was aimed to evaluate the ability of BIA use during pregnancy to predict the development of pre-eclampsia. It has been observed that BIA measurement applied to pregnant women at 17–20 weeks of gestation has good accuracy and a high negative predictive value for the risk of pre-eclampsia development [41]. In other study conducted by Staelens et al., it was aimed to evaluate the changes in maternal body fluid composition during uncomplicated pregnancy and the differences between uncomplicated and hypertensive pregnancies by BIA analysis. Measurements showed that total body water, ICW, ECW, and ECW/ICW ratio increased significantly during pregnancy and the ECW/ICW ratio was higher in preeclamptic patients [42].

### Dual Energy X-Ray Absorptiometry

While Dual Energy X-ray Absorptiometry (DEXA) is not suitable in pregnancy because of radiation exposure, DEXA can be used in the pre-and post-pregnancy periods to measure bone mineral content, which is a component in the four-compartment model estimation of body composition and can also be used for adipose and lean tissue estimations [3, 43].

### Ultrasound

Ultrasound is a technique that involves the production of sound waves of varying frequencies to measure the adipose tissue during pregnancy. The skinfold thickness estimates obtained by ultrasound correlate highly with the estimates made by the caliper. However, ultrasound predictions were found to be superior with increased accuracy and precision. Also, ultrasound eliminates the need for pressure when using a caliper when measuring skinfold thickness [44]. However, no studies have validated this method against other methods such as magnetic resonance imaging (MRI), which would be considered the gold standard [1].

In the study conduct by Benevides et al., abdominal adipose tissue was evaluated by ultrasound measurements for predicting gestational diabetes mellitus (GDM) in the first and second trimesters of pregnancy. It was stated that the use of a threshold of 45.25 mm for maximum preperitoneal fat measured by ultrasound for early estimation of GDM risk, especially in non-obese pregnant women, be like a suitable, inexpensive, and practical alternative to be included in clinical practice in the first trimester of pregnancy [45]. However, standard protocols for assessing body fat by ultrasound have not been developed. Therefore, reliable and valid monitoring of adipose tissue changes throughout pregnancy may be difficult with this method [44, 46, 47]. As ultrasound is widely available in hospital, more research is needed to develop standardized protocols for measurement and analysis of their validity and reliability [3].

### Computed Tomography and Magnetic Resonance Imaging

Computed tomography (CT) and MRI can be used to estimate body composition. However, there is not enough data on the use of magnetic resonance in pregnancy. Computed tomography is contraindicated due to radiation exposure and is not used to evaluate changes from pre-pregnancy to the postpartum period [48].

The magnetic resonance imaging method is an in vivo imaging technique that uses a strong magnetic field to measure tissues and organs. MRI is useful for body composition because it can yield results such as adipose tissue and skeletal muscle mass, and individual organ sizes with whole-body or regional scanning protocols [49, 50]. Fetal MRI is a significant diagnostic imaging technique like ultrasonography, particularly for evaluation of the fetal brain, lungs, and bowel, as well as the placenta [51].

MRI is the only method available for the in vivo measurement of adipose tissue, skeletal muscle, and organ mass that can predict changes in body mass and distribution throughout pregnancy. However, density and hydration cannot be measured by MRI. MRI is used in maternal conditions such as placental abruption, ovarian cysts, acute abdominal pain, acute appendicitis, pancreatic and bile duct pathology, pregnancy-related spine problems, neurological conditions and monitoring of multiple sclerosis in pregnancy. For the fetus, MRI is used in fetal indications such as the central nervous system and congenital anomalies [49].

Although MRI research is currently the most modern method available in pregnancy, MRI measurement has several limitations, including cost, technician expertise, unsuitable for field studies, the unwillingness of pregnant women to be exposed to magnetic fields, and the time required for testing and analysis [3, 51]. Our knowledge of MRI safety during pregnancy and the application of gadolinium-based contrast agents that can cross the placental barrier is limited, raising concerns about possible adverse effects on both maternal and fetal health. It is preferable to use contrast chemicals that cannot safely pass through the placenta. Although the safety of these novel contrast agents has been assessed in human model studies, more research is required to fully elucidate their possible crucial function and to validate their safety during pregnancy [49, 52].

In a cohort study conducted by Ray et al. evaluated the fetal and childhood outcomes of MRI during the first trimester of pregnancy or exposure to gadolinium at any time during pregnancy. Magnetic resonance applied in the first trimester was not significantly associated with stillbirth or neonatal death, congenital anomalies, neoplasms, or hearing loss, and Gadolinium-enhanced magnetic resonance was associated with a higher risk of stillbirth or neonatal death, and a range of rheumatologic and inflammatory skin conditions. Further studies are needed to address the different limitations of the study and to replicate these findings [53].

Anthropometric measurements and laboratory methods used in the evaluation of body composition in pregnancy are summarized in Table 4 and 5.

**Table 4:** Anthropometric measurements and laboratory methods used in the evaluation of body composition in singleton pregnancy

Type of Measurement	Country	Aim	Participant Characteristics	Relevant Outcomes	Reference
**Anthropometric Measurements**					
Gestational Weight Gain	Korea	Impact of pre-pregnancy BMI and GWG on the risk of maternal and infant pregnancy complications	2454 pregnant women (Mean age: 33.3 ± 3.8 years Gestational age: 275.0 ± 10.0 days)	-Women with excessive weight gain had a 36.4% increase in the risk of adverse pregnancy outcomes -Women with overweight or obesity before pregnancy and had excessive GWG had a three-fold increase in the risk of adverse pregnancy outcomes	[54]
	USA	To evaluate GWG and change in fat mass from early (13–16 weeks) to late (35–37 weeks) pregnancy	54 singleton pregnant women with obesity (BMI ≥ 30.0 kg/m^2^)	- The composition of GWG differs in each trimester. In the second trimester, GWG consists largely of fat mass accumulation, while in the third trimester, GWG consists mostly of lean mass and fetal tissues	[55]
Skinfold Thickness	Ireland	To evaluate maternal body composition parameters for develop a prediction model for GDM in early gestation	188 pregnant women aged between 18 and 50 years (Gestational age: Between 10 and 16 weeks)	-GDM developed in 20 of 188 women underwent OGTT at the 28th week of pregnancy -The truncal skinfold thickness (supraspinal + subscapular + abdominal + iliac crest) was significantly higher in women with GDM compared to non-GDM	[56]
	USA	To examine how changes in anthropometric parameters (MUAC, and skinfolds of the triceps, thigh, and upper iliac) at < 12, 16–22, and 28–32 weeks of gestation	360 pregnant women (Mean age: 30.6 ± 4.5 years)	- MUAC, triceps, and thigh skinfolds, and fat gain were all inversely associated with inadequate GWG, whereas all parameters were positively associated with excessive GWG	[57]
	India	To evaluate the effect of gestational glucose intolerance (GGI) in pregnancy on anthropometry and adiposity of their newborns	119 pregnant women and their newborns	-Gestational glucose intolerance during pregnancy does cause increased adiposity in their newborns -The triceps and subscapular skinfold thicknesses which are direct measurements of adiposity were significantly higher in newborns of GGI mothers compared to newborns of GDM and non-glucose intolerance mothers	[58]
MUAC	India	To identify the appropriate substitute parameter for skinfold thickness to estimate total body fat in pregnant women and their newborns	3720 pregnant women and their newborns (Gestational age: Between 14 and 36 weeks)	MUAC (> 29.2 cm) and birth weight (> 3.4 kg) can be used as suitable alternative for skinfold thickness, reflecting total body fat and obesity in pregnant women and their newborns, respectively	[59]
	Sudan	To compare MUAC with BMI and propose MUAC cut-off points corresponding to BMIs of < 18.5 kg/m^2^ and ≥ 30.0 kg/m^2^ for pregnant women	688 pregnant women (Mean gestational age: 17 weeks)	-There was a significant positive correlation between BMI and MUAC -The cut-off points of MUAC for detecting underweight and obesity were found to be 24.0 cm and 29.0 cm, respectively, for women in early pregnancy (< 20 weeks of gestation) - For women in late pregnancy, the cut-off points were 23.0 cm and 28.0 cm, respectively (≥ 20 weeks of gestation)	[60]
**Laboratory Methods**					
Total Body Water *(Isotope Dilution Tecnique)*	India	To evaluate the association of maternal TBW measured by the deuterium dilution technique (D_2_O) at 17 and 34 weeks of gestation with birth weigh	99 pregnant women (Rural n = 51, urban n = 48) (17 and 34 weeks of pregnancy)	-They drank 75 mg/kg body weight of deuterated water and urine samples were collected at the fifth and sixth hours after drinking the deuterated water -Only rural women between 17 and 34 weeks had a greater birth weight when their dry mass (weight minus TBW-D_2_O) increased or decreased as a percentage of total weight	[61]
	Kenya	To examine the relationship between maternal body composition and infant birth weight	129 pregnant women (Mean age: 23.8 + 5.2) (First or second trimester of pregnancy)	-Deuterium dilution technique was used to determine body composition, including TBW, FFM and FM -There was a significant relationship between birth weight and maternal total body water and fat-free mass, no significant relationship was found between fat mass	[62]
Body Density *(ADP-BOD POD)*	Sweden	To compare the measured and predicted TGV of pregnant women in gestational week 32	27 pregnant women (Mean gestational age: 32 weeks)	- Body weight and body volume of the pregnant women were measured using BOD POD and the predicted thoracic gas volume was on average 6% higher than the measured gas volume	[34]
	Finland	To investigate the effect of measuring versus predicting TGV on the accuracy of body composition calculations measured with ADP in overweight and obese pregnant women	110 pregnant women (Mean gestational age: 35 weeks)	-Using predicted instead of observed TGV in body composition calculations resulted in an overestimation of fat mass during the early and late stages of pregnancy -Measuring TGV during the late stage of pregnancy in women with overweight and obesity increases the accuracy of body composition measurement by ADP	[63]
BIA	Italy	To evaluate hypertensive disorders in pregnancy among women with obesity through the BIA at the first trimester	479 pregnant women with obesity (Mean gestational age: 9–12 weeks)	- There were significant differences in body composition between the two groups (hypertensive and normotensive) - From the first trimester, women with developing gestational hypertension showed higher values of total body mass, FM, FFM, TBW, and leg FM, FFM - Women with high TBW levels in the first trimester were found to have a higher risk of developing gestational hypertension	[64]
	Australia	To evaluate the repeatability of three BIA devices (Bodystat 1500, RJL Quantum III, and Tanita BC-587) in pregnancy and analyze the relationship between the body composition and the GDM risk	117 pregnant women (Mean age: 33.1 ± 4.9)	-In all three trimesters, the repeatability between Bodystat and RJL was stronger than that between Bodystat and Tanita in terms of body fat% and TBW% - RJL overestimated body fat% on average by 3.3% -Average body fat% was not significantly different between Tanita and Bodystat	[65]
BIVA	Italy	To identify body composition via BIVA variables (R/H, Xc/H, Z/H) during physiological pregnancy	37 pregnant women (Mean age: 31.95 ± 4.02)	- There was a significant decrease in BIVA variables and a shortening of the vector, indicating that TBW and hydration increased significantly - The vector length increased significantly, indicating a physiological gain of FM%. No changes were found for the phase angle, indicating that the ECW/ICW ratio remained unchanged - The values of BIVA between 12th–13th and 30th–31st week were low, but in the postpartum period, they tended to be similar to those recorded at the beginning of pregnancy	[66]
Ultrasound	Romania	To evaluate the efficacy of umbilical cord biometry and fetal abdominal skinfold evaluation as screening tools for fetal macrosomia in pregnant women with GDM	51 pregnant women with GDM (Mean age: 32.5 ± 4.95)	-There was a statistically significant difference in the abdominal skinfold measurement during the second trimester between macrosomic and normal-weight newborns in the GDM patient group -In the GDM patient group, the second-trimester abdominal circumference had a statistically significant correlation with fetal macrosomia at term -Abdominal skinfold measurement and abdominal circumference during the second trimester may be significant markers of fetal metabolic status in GDM-complicated pregnancies	[67]
MRI	Israel	To assess the long-term neurodevelopmental outcomes of childrens exposed to MR imaging during pregnancy	131 pregnant women (Mean gestational age at birth: 39 weeks)	-Long-term neurodevelopmental outcomes were evaluated of their children, 2.5 to 6 years of age -The use of 1.5 T noncontrast MR imaging during pregnancy had no negative consequences on long-term neurodevelopmental results	[68]
	Turkey	To investigate congenital central nervous system (CNS) abnormalities by MRI in pregnancy	110 pregnant women (Mean gestational age 26.5 weeks)	-There were no statistically significant differences between the accuracy of fetal neurosonography and fetal MRI for fetal anomaly groups. Fetal MRI may improve diagnostic accuracy in pregnancies with congenital CNS abnormalities	[69]
Multiple Methods	USA	To compare several approaches for estimating FM in pregnant women, including 2-, 3-, and 4-compartment models, and to specify how these measures compare to FM measured DEXA 2 weeks postpartum	41 pregnant women (Mean gestational age: 37 weeks)	- From lowest to highest, the following methods were used to estimate fat mass: 4- compartment model, DEXA, TBW, 3- compartment model, BIA, body density by ADP, and skinfold thickness - Body density by ADP 3-compartment models had the highest precision	[36]
Multiple Methods	Sweden	To compare body composition by BIA and MRI to pregnancy-adjusted ADP, as well as FM changes during pregnancy	151 normal weight and obese pregnant women (Age: 20–45 years)	- MRI and ADP measured fat mass increases in women with obesity similarly, while BIA overestimated fat mass changes in normal-weight women - In women with obesity, the mean FM and FM changes measured by MRI and pregnancy-adjusted ADP were similar - In normal-weight women, mean fat mass by QMR and fat mass changes by BIA were higher than the comparable values determined by pregnancy-adjusted ADP	[70]
Multiple Methods	Canada	-To evaluate changes in between % body fat, skinfold thickness and BIA during pregnancy and in postpartum; -To evaluate relationship between skinfold thickness and BIA with DEXA in the postpartum	181 pregnant women (Mean gestational age: 13 weeks)	- In early pregnancy, % body fat by skinfold thickness and BIA showed strong agreement, with BIA values 1.8% higher than skinfold thickness, but low agreement in late pregnancy, with BIA values 7.1% greater than skinfold thickness	[71]

ADP: Air Displacement Plethysmograph, BIA: Bioelectrical Impedance Analysis; BIVA: Bioelectrical Impedance Vector Analysis; BMI: Body Mass Index CT: Computed Tomography; DEXA: Dual Energy X-Ray Absorptiometry; ECW: Extracellular Water; FM: Fat Mass; FFM: Fat-free Mass; GGI: Gestational Glucose Intolerance; GDM: Gestational Diabetes Mellitus, GWG: Gestational Weight Gain; ICW: Intracellular Water; MUAC: Middle Upper Arm Circumference; MRI: Magnetic Resonance Imaging, OGTT: Oral Glucose Tolerance Test, USA: United States of America, TBW: Total Body Water, TGV: Thoracic Gas Volume

**Table 5:** Anthropometric measurements and laboratory methods used in the evaluation of body composition in twin pregnancies

Type of Measurement	Country	Aim	Participant Characteristics	Relevant Outcomes	Reference
**Anthropometric Measurements**					
Gestational Weight Gain	China	To investigate maternal and neonatal outcomes of twin pregnancies complicated by gestational diabetes mellitus	897 women with GDM twin pregnancies (Mean age: 31.40 ± 3.6 years)	-Maternal age ≥ 35 years, overweight or obesity, and chronic hypertension before to pregnancy were significant risk factors for GDM in twin pregnancies	[72]
	USA	To estimate the pregnancy outcomes in overweight and obese women with twin pregnancies	252 overweight and obese women with twin pregnancies (Mean age: 34.6 ± 6.8 years)	- Women with inadequate average gestational weight gain had significantly higher rates of spontaneous preterm birth < 37 weeks, a higher likelihood of any twin birth weight < 10th percentile for gestational age and lower birth weights for both larger and smaller twins, - For overweight and obese women with twin gestations, meeting the IOM recommendations for weight gain n pregnancy is associated with improved pregnancy outcomes	[73]
BMI	Canada	To test the relationship between maternal pre-pregnancy BMI and adverse pregnancy outcomes differ between twin and singleton gestations	487,870 women with singleton and twin pregnancies	- In both singleton and twin pregnancies, high maternal BMI increased the risk of main outcomes like preeclampsia, gestational diabetes, and cesarean birth - In women with twin pregnancies, underweight is associated with the greatest risk for preterm birth	[74]
Gestational Weight Gain and BMI	Germany	To investigate maternal weight, gestational weight gain and associated outcomes in twin pregnancies	10,603 women with twin pregnancies (Mean age: 32.1 ± 5.1)	- Rates of birthweight < 1500 g and of preterm birth < 28 and from 28 to 33 + 6 weeks all increased -No significant changes were observed in the rates of stillbirth, perinatal mortality and neonatal intensive care unit admissions	[75]
Skinfold Thickness	Germany	To investigate the longitudinal assessment of fetal biometry and abdominal fat thickness of twin pregnancies	30 women with dichorionic-diamniotic twin pregnancy	-The rise in fetal subcutaneous adipose tissue thickness curves was slower in the twin offspring of women with history of gastric bypass as compared to the offspring of obese mothers and offspring of BMI-matched controls	[76]
**Laboratory Methods**					
Ultrasound	Canada	To compare via ultrasound fetal growth pattern between twin and singleton fetuses	543 women with twin pregnancies (Mean age: 33.8 ± 5.2 years)	- Estimated weight of twin fetus emerged as lower than that of singletons starting at 26 weeks of gestation, and these differences increased with gestational age, reaching a mean difference of 300–350 g at term -Twin fetus experience slowing of growth beginning at ∼26 weeks of gestation and a greater degree of asymmetric growth pattern compared with singletons	[77]
MRI	Canada	To investigate of fetal cerebral ischemic injury in monochorionic twin pregnancy complicated by spontaneous single intrauterine death using MRI	47 women with monochorionic twin pregnancy (Mean age: 30.5 ± 5.1 years)	-MRI has important roles in early imaging of fetal cerebral ischemic injury in monochorionic twin pregnancy	[78]
	Italy	To describe fetal brain MRI fndings in monochorionic pregnancies complicated by TAPS prenatally diagnosed, and to characterize the potential intracranial complications associated with this condition and potential treatment options	29 women with monochorionic twin pregnancy (Mean maternal age: 22 weeks)	-MRI has important roles in imaging anomalies such as hemorrhagic lesions, brain edema-swelling and venous congestion in twin pregnancies	[79]
Multiple Methods	USA	To evaluate the relationship of maternal body composition changes on birth weight, birth length, and neonatal FM in dichorionic-diamniotic twin pregnancies	20 women with twin pregnancies (Mean age: 32.3 ± 4.0 years)	-Maternal FM change from the second to the third trimester was significantly correlated to neonatal FM percentage -Third trimester GWG and total protein gain were positively correlated with neonatal birth length -Significant increases in maternal protein are associated with greater birth weight and neonatal birth length. Protein accretion, in contrast to TBW and FM gains, may be the most critical component of maternal GWG in dichorionic twin gestations	[80]

ADP: Air Displacement Plethysmograph, BIA: Bioelectrical Impedance Analysis; BIVA: Bioelectrical Impedance Vector Analysis; BMI: Body Mass Index; FM: Fat Mass; GDM: Gestational Diabetes Mellitur; GWG: Gestational Weight Gain; MUAC: Middle Upper Arm Circumference; MRI: Magnetic Resonance Imaging, TAPS: Twin Anemia-Polycythemia Sequence; TBW: Total Body Water; USA: United States of America

## Conclusion

Accurate assessment of anthropometric measurements and body composition in pregnant women is an important technique in revealing interactions between fat mass and the health of the mother and infant. Anthropometry (skinfold thickness and middle upper arm circumference) is the most used method to measure maternal body composition changes during pregnancy. Various body composition equations have been developed using skinfold thickness measurements during pregnancy. Methods based on densitometry and hydrometry provide important estimates of adipose tissue and lean tissue, but adjustment for changing lean tissue hydration during pregnancy is required. Although BIA has been used in studies in the literature, it does not provide a complete measure of maternal body composition. The inability to use methods such as CT and DEXA due to ionizing radiation, the high cost of imaging techniques such as nuclear magnetic resonance, and the low accuracy of BIA are the main factors that limit the evaluation of body composition during pregnancy. However, with DEXA just before pregnancy and after birth measuring maternal body composition remains valuable for understanding changes in body composition during pregnancy.

With the developments in today's technology, imaging technologies have begun to shed light on important points in the evaluation of body composition during pregnancy. It is thought that the increased tendency to MRI during pregnancy will make it easier to understand the changes in fat distribution and provide the opportunity to evaluate the mother's body composition separately from the body composition of the fetus. MRI is commonly used in pregnant wome, most commonly to assess, acute pelvic and abdominal pain, neurological abnormalities, as well as placental or fetal abnormalities, neoplasms and infections. However, the use of these approaches remains limited due to cost, need for expert personnel and equipment, and safety concerns that prevent evaluation during pregnancy.

Most studies in the literature have been conducted with women with singleton pregnancy. Because women with multiple pregnancies are often excluded from studies, more studies are needed to examine changes in body composition in women with multiple pregnancies. Expanding the available data on body composition in pregnant women will allow a better comparison of energy intake and expenditure and a better estimation of energy requirements for healthy fetal growth.

In summary, in the light of all this information, maternal body composition changes throughout pregnancy in order to support healthy growth of the fetus. The amount and content of healthy gestational weight gain differ significantly differs for women who are underweight, normal weight, overweight, and obese, and it is inversely connected to the pre-gravid BMI class. In cases where pre-gestational weight measurement and subsequent BMI values are unavailable, anthropometric measurements, MUAC, and skinfold thickness are useful and may be ideal for measuring pregnant women's nutritional status, particularly in low- and middle-income countries. Future research should focus on improving the prediction of changes in body composition throughout pregnancy, particularly pre-pregnancy BMI and body composition, and improving maternal and child health during and after pregnancy should be the primary goal.

## Data Availability

No datasets were generated or analysed during the current study
